# Social media use and mental health of urban residents during China's second COVID-19 outbreak

**DOI:** 10.3389/fpubh.2022.1016849

**Published:** 2022-12-08

**Authors:** Zhenhua Zheng, Ning Sun, Yu Chen, Hong Chen

**Affiliations:** ^1^College of Communication and Art Design, University of Shanghai for Science and Technology, Shanghai, China; ^2^School of Economics, Sichuan University, Chengdu, China; ^3^College of Architecture and Environment, Sichuan University, Chengdu, China

**Keywords:** social media, COVID-19, mental health, depression, anxiety

## Abstract

**Background:**

A multitude of literature has shown that during the 2019 COVID-19 outbreak, people's reliance on social media has been closely related with serious psychological problems. The “information epidemic” has sparked each country's attention. These countries including China have tried to find the solution and taken a series of measures. In January 2021, the COVID-19 broke out again in Shijiazhuang, China. Has the impact of social media on mental health changed?

**Methods:**

Our data are based on an online survey of Chinese in January 2021, with 904 valid samples from 18 different provinces in China. We applied the methods of structural equation model analysis and the tendency value matching to conduct systematic analysis.

**Results:**

Our research found that 38.9% of the population suffered from depression and 12.61% of the population suffered from anxiety. Chinese urban residents are more dependent on social media, with up to 80.1% of participants using social media frequently. Our research found that the relationship between social media use and residents' mental health has dramatically changed. More use of social media has been significantly associated with less depression and anxiety, especially among young people and women. Our findings are the first to reveal the relation's change between social media and mental health.

**Conclusions:**

These findings implied that changes in the social media environment probably lead to changes in relationship between social media use and mental health since the outbreak began in 2019. Truthful and comprehensive social media information and a healthy positive social media environment can contribute to residents' mental health improvement and the fight against “information epidemic.”

## Introduction

The COVID-19, which began in December 2019, has swept the world, causing not only huge losses to the lives and economies of all mankind, but also seriously affecting the mental health of ordinary people (1, 2). The COVID-19 outbreak was first detected in December 2019 in Wuhan, and China was the first country affected by the coronavirus pandemic, thus the development and reply of China's COVID-19 epidemic has been concerned internationally (3, 4).

After March 2020, in China, the epidemic of COVID-19 was well under control and people's lives gradually returned to normal. A follow-up study of the mental health of Chinese residents found that the levels of stress, anxiety and depression had been relatively stable since March 2020 (5). However, in January 2021, China's COVID-19 partially reoccurred in Shijiazhuang, Hebei Province. China once again launched war-time control measures: the whole city was under closure management; airports, railways and public transport were all suspended; about 10.25 million residents were quarantined at home, and on January 7, 12, 20 all residents carried out three nucleic acid tests. More than 20,000 people were in a centralized isolation during this outbreak. Since China was deemed to have “defeated the COVID-19 epidemic,” the second round of the local concentrated outbreak dragged the Chinese from bystanders to face the pandemic. The pressure and anxiety associated with COVID-19 have become apparent.

After the global outbreak of the COVID-19 in 2019, the media is the main source of information about COVID-19 (6), especially social media (7, 8). China's major social media sites have a dedicated “COVID-19 Fight” section. WeChat added more than 100 “mini-programs” related to COVID-19, updating information related to the outbreak simultaneously. In addition, many self-media and netizens also published and disseminated relevant information on WeChat, Weibo, and other social media (9).

Thus, the impact of social media on people's mental health has been highly concerned by scholars, governments and the International Health Organization (10). The research conclusions of the existing literature are consistent, that is, more reliance on social media has led to severe depression, anxiety, and other psychological problems after the outbreak (3, 11–13). Since the pandemic began in 2019, social media has been bombarded with false information and reports about COVID-19, sparking groundless fears among many netizens (14, 15). A large number of gossips and misinformation pose a serious threat to public health (16, 17). The COVID-19 epidemic has developed into an unprecedented “information epidemic” (11).

In fact, prior to the outbreak in 2019, the support of social media for people's mental health was confirmed by many scholars. Researches by some scholars have suggested that social media can provide users with affluent information, help obtain solutions to problems such as doubts, health crises, etc., and help improve emotional threats and protect mental health (18, 19). What's more, social networking provides valuable emotional support, through which users can feel being accepted, loved and respected (20), a sense of belonging and self-affirmation (21), which in turn improves mental health issues (22).

Therefore, we found that the impact of social media on people's mental health varies among different social contexts and media environments, or even the complete opposite. As a result, in such a special social environment as CODIV-19 outbreak, it is necessary to explore the relationship between social media use and the mental health of residents in different social media environments. In any case, as some scholars have pointed out, the significant impact of social media on people's mental health has been widely recognized. Governments of countries and health departments are struggling to find effective solutions to the “information epidemic.” While the Chinese government has taken strong measures to control the COVID-19, it has also stepped up its efforts to govern the social media environment. On April 16, 2020, the National Health Council of China issued guidelines for local governments to promote psychological crisis intervention for residents during public health emergencies. However, after the social media environment has changed, it remains unclear whether the mental health problems of Chinese residents during another local outbreak in China, and whether the impact of social media on mental health will change at this time. Therefore, there is a need for a rapid assessment of the factors affecting the mental health during the second outbreak (23).

In addition, numerous studies have also focused on the heterogeneity of depression that occurred during the pandemic (24). For example, during the period, the prevalence of depression in women was significantly higher than that in men (5, 11, 25), and young people under 40 are more susceptible to depression (26–30). However, so far the impact of social media on public psychology during the COVID-19 pandemic has received due attention, while the heterogeneity of how social media use can affect mental health remains almost unnoted and how it differently works on the disadvantaged and the advantaged remains unclear.

Based on the above-mentioned current Chinese situation, as well as the literature review and analysis, our research raises the following questions:

1. What are the mental health problems of ordinary Chinese during the second round of COVID-19 outbreak in January 2021? Is there any heterogeneity?
2. During the second round of the COVID-19 outbreak, what is the situation of the use of traditional media and social media?
3. Is there any difference of the relationship between social media us and mental health among different social groups during the second round of COVID-19 outbreak?
4. Has the relationship between social media use and mental health such as depression and anxiety changed after the Chinese government took a series of measures to control the social media environment?

## Methods

### Design and participants

The data of the study were cross-sectional data from the online survey, and the questionnaires were distributed by non-directional random, with participants aged 18 and older. We surveyed urban residents of Shijiazhuang who were living in isolation at home and those who had restricted mobility outside Shijiazhuang. The survey was conducted between January 10 and January 15, 2021, 1 week after the closure and home isolation in Shijiazhuang during the second round of China's COVID-19. The survey was conducted through the questionnaire star platform (https://www.wjx.cn/app/survey.aspx), which invited Chinese urban residents to participate online. All participants in the survey volunteered to fill out the questionnaire, which was conducted under the supervision of the Academic Committee of University of Shanghai for Science and Technology. All participants in the survey were asked to answer specific questions about demographics, social media use, depression, anxiety, and other changes in mental health. In the questionnaire design, we set multiple restrictions to ensure the validity of the data. Specifically include: Filtering for duplicates of IP. 1. Duplicate questionnaires from the same device, IP address or Wechat account were deemed invalid. 2. Time limit for answering questions. Questionnaires that take <150 s to fill out were considered invalid. 3. The design of questionnaire questions. The questionnaire was occasionally interspersed with basic cognitive questions, including “Where is the capital of China?”, “Which picture is a square?”, “3 + 6 = ?”. If one of the above questions is answered incorrectly, the questionnaire will be considered invalid. Once the questionnaire is valid, the participant will receive a bonus (¥ 5 per person, approximately $ 0.78). We finally screened out 904 valid samples from the 1,204 samples, including 412 samples from Shijiazhuang residents who were isolated at home and 492 samples from residents with restricted mobility in other areas. As of this writing Shijiazhuang is still under lockdown, during which the psychological health of residents is uncertain, so it is particularly important to obtain rapid and timely data. Although the network survey is not a rigorous sample survey, the sample representation is not perfect, but the advantage of the network survey is the rapid access to data in the event of emergency. The distribution of the sample population is shown in Table 1. From the statistical results of valid samples, although it is not completely consistent with the distribution of China's population, it also covers the majority of groups. Therefore, our sample has some representative significance.

**Table 1:** The sample demographics.

**Demographics**	***N***	**%**
**Overall**	904	(100)
**Age**		
39–	412	45.58
40–59	402	45.46
60+	72	7.96
**Gender**		
Male	320	35.4
Female	584	64.6
**Education**		
Junior high school and under	58	6.42
Senior high school; technical and vocational schools	74	8.19
Junior college (with associate degrees)	208	23.01
Undergraduate	398	44.03
Master and above	166	18.36

### Measurement

#### Mental health

According to previous studies, depression and anxiety assessment have become the most important measurement of mental health. Depression is assessed through a Chinese version of depression by The Chinese version of WHO-Five Well-Being Index (WHO-5), including five positive emotional items: (1) feeling happy and comfortable, (2) feeling calm and relaxed, (3) feeling energetic, (4) feeling sober after waking up, getting enough rest, (5) every day's life is abound with interesting things. Participants were asked how often they had these positive emotions since the COVID-19 outbreak. A score of 6 points was used, from all times (5) to no time (0). Less than 13 scores indicate depression.

Anxiety is assessed with the widely used Anxiety Scale GAD-7 (11, 31), including 7 negative items: (1) feeling nervous, worried and anxious, (2) being unable to stop or control worry, (3) worrying too much about all kinds of things, (4) being difficult to relax, (5) being unable to sit still due to restlessness, (6) becoming prone to trouble or impatience, (7) feeling that something terrible would happen and scared. Participants were asked how often they had these negative emotions since the COVID-19 outbreak. Response options are “not at all,” “less than half the time,” “more than half the time,” and “almost daily,” with assignments of 0, 1, 2, and 3, respectively. A total score of more than 10 represents that the respondents suffers from anxiety.

#### Social media usage

Social media use is done by asking respondents how often they have accessed information about COVID-19 through social media since the outbreak (11). In order to more accurately reveal the impact of social media on mental health, we included both social media and traditional media as independent variables in the model to compare their effects on mental health. Traditional media mainly include television, radio, newspapers, official websites, magazines and so on. Social media include WeChat, Weblog, Zhihu, Douyin, Toutiao, news networks and so on. The response is measured by using the five-point Likert scale of the “never” (1), “occasionally” (2), “sometimes” (3), “often” (4), and “always” (5).

#### Covariates

The regression model is adjusted according to a number of personal factors, including income, education (1: junior high school and below; 2: senior high school, secondary school and technical school; 3: college; 4: undergraduate; 5: master degree and above), gender, age, marital status (0: in marriage; 1: not married), number of people living together, years of residence, self-rated health (SRH) (1: very bad; 2: poor; 3: General; 4: Better; 5: Good).

First, our model controlled variables that may be related to social classes, such as income and education. The rationale behind is that social classes are found to be associated with mental health (32, 33). Second, as a large number of literatures have identified the effects of living conditions on mental health (34), we controlled two variables: the number of people living together and the number of years of residence to increase robustness of the model. Finally, we controlled SRH, because SRH has a direct impact on emotional health, covariate to SRH, but also help to increase the robustness and effectiveness of the model.

#### Statistical analysis

This study selects descriptive statistical analysis and structural equation model (SEM) for statistical analysis.

In order to test whether the data is suitable for the method of SEM, we have significant t-checking of all observed variables, which are split by 27 and 73 scales, and the results show that all variables have good identification. The factor verification analysis of depression and anxiety measurement model is carried out. The results showed that the component reliability of the measurement model was >0.6, the average variance extraction was >0.5, the factor load of the observation variables was >0.6, and the reliability coefficient was >0.36 (35), which showed that the measurement model all has good reliability and validity, and were suitable for SEM analysis. The results of depression model and anxiety model fitting showed that the fit index GFI value, RMSER, card-square degree of freedom ratio (X2/DF), AGFI value, IFI value and CFI value all meet the ideal standard, and the model has good fitness.

In addition, our study is based on cross-sectional data, so in order to explain the relationship between social media and residents' mental health as much as possible under limited conditions, and to avoid the effects of confounding effects as much as possible, we used Propensity Score Matching (PSM) to analyze and verify the robustness of the structural equation model results.

PSM achieves a random assignment effect by matching the samples of the treatment group and the control group one by one to control the self-selecting mechanism interference caused by observable variables, and by controlling the propensity value, it is possible to “approximately” satisfy the non-obfuscation assumption under the statistical counterfactual framework and thus make causal inferences (36). The basic process of PSM is to estimate the probability of each sample being grouped into a processing group by using the Logit model according to the observable confusion variables, obtain its propensity score, and then match the samples with the closest propensity values but belonging to the two groups one by one. We used proximity matching to obtain a design effect similar to that of randomized trials. Finally, the average treatment effect on treatment (ATT) and significance of social media on depression and anxiety in residents were obtained.

## Results

### Descriptive statistics for the sample

#### Mental health

##### Depression

Depression symptoms were present in 38.9% of the overall sample. Age 39– (40.8%), female (41.4%), master's degrees and above (43.4%), low income (40.1%), unmarried (43.0%), students (41.8%), poor SRH (61.9%), number of people living together 4+ (42.4%), and years of residence 4– (40.2%) had a higher prevalence of depression, all by more than 40%.

##### Anxiety

12.6% of the overall sample had anxiety symptoms. Age 39– (13.1%), female (12.7%), college and below (18.8%), low income (15.8%), unmarried (19.4%), students (16.4%), poor SRH (21.6%), number of people living together 4+ (16.0%), and years of residence 5+ (12.8 %) were more likely to have higher anxiety.

It is worth noting that such groups as Age 39–, female, low income, unmarried, students, poor SRH, the number of co-residents 4+ reflect a higher prevalence of depression and anxiety. See Table 1 for details.

Through the comparison of model results, we found no significant difference in the association between social media use and mental health among Shijiazhuang residents living in isolation and those living in other areas.

#### Social media usage

##### Traditional media

In the overall sample usage frequency, the proportions of choosing “less,” “sometimes,” and “often” were 24.3, 18.1, and 57.5%, respectively. The groups, including middle-age and above, college and below, local personnel, very good SRH, use traditional media significantly more than others. The frequency of using traditional media in these groups is 51.9, 63.5, 60.3, and 65.1%, respectively.

##### Social media

In the overall sample usage frequency, the percentages of “less,” “sometimes,” and “often” were 10.8, 9.1, and 80.1%, respectively. Groups, including Age 39–, master's degrees and above, high-income, students, and local people groups, use social media significantly more frequently than other groups, which frequency of using social media is in order: 81.6, 89.2, 88.6, and 81.8%. See Table 2 for details.

**Table 2:** Demographic distribution of traditional and social media usage in the sample.

**Demographics**	**Traditional media use** ***N*** **(%)**			***P***	**Social media use** ***N*** **(%)**			***P***
	**Less**	**Sometimes**	**Often**		**Less**	**Sometimes**	**Often**	
**Overall**	220 (24.3)	164 (18.1)	520 (57.5)		98 (10.8)	82 (9.1)	724 (80.1)	
**Age**								
39–	108 (26.2)	90 (21.8)	214 (51.9)	0.015	38 (9.2)	38 (9.2)	336 (81.6)	0.196
40+	112 (22.8)	74 (15.0)	306 (62.2)		60 (12.2)	44 (8.9)	388 (78.9)	
**Gender**								
Male	80 (25.0)	46 (14.4)	194 (60.0)	0.519	40 (12.5)	24 (7.5)	256 (80.0)	0.553
Female	140 (24.0)	118 (20.2)	326 (55.8)		58 (9.9)	58 (9.9)	468 (80.1)	
**Education**								
College and below	82 (24.1)	42 (12.4)	216 (63.5)	0.060	52 (15.3)	32 (9.4)	256 (75.3)	0.000
Undergraduate	94 (23.6)	78 (19.6)	226 (56.8)		40 (10.1)	38 (9.5)	320 (80.4)	
Master's degree and above	44 (26.5)	44 (26.5)	78 (47.0)		6 (3.6)	12 (7.2)	148 (89.2)	
**Household per capita income (Yuan/Month)**								
5,000–	76 (25.0)	50 (16.4)	178 (58.6)	0.226	38 (12.5)	28 (9.2)	238 (78.3)	0.000
5,001–12,000	84 (22.6)	64 (17.2)	224 (60.2)		54 (14.5)	34 (9.1)	284 (76.3)	
12,001+	60 (26.3)	50 (21.9)	118 (51.8)		6 (2.6)	20 (8.8)	202 (88.6)	
**The state of marriage**								
Married	170 (23.7)	124 (17.3)	424 (59.1)	0.125	80 (11.1)	68 (9.5)	570 (79.4)	0.367
Unmarried	50 (26.9)	40 (21.5)	96 (51.6)		18 (9.7)	14 (7.5)	154 (82.8)	
**The person property**								
Students	28 (25.5)	32 (29.0)	50 (45.5)	0.028	10 (9.1)	10 (9.1)	90 (81.8)	0.006
Local employees	168 (23.3)	118 (16.4)	434 (60.3)		76 (10.6)	56 (7.8)	588 (81.8)	
Personnel from the field	24 (32.4)	14 (18.9)	36 (48.6)		12 (16.2)	16 (21.6)	46 (62.2)	
**SRH**								
Very good	56 (22.2)	32 (12.7)	164 (65.1)	0.099	26 (13.4)	24 (12.4)	144 (74.2)	0.130
Better	112 (24.6)	100 (21.8)	246 (53.7)		44 (9.6)	42 (9.2)	372 (81.2)	
General/poor/very poor	52 (26.8)	32 (16.5)	110 (56.7)		28 (11.1)	16 (6.3)	208 (82.5)	
**Number of people living together**								
1–2	64 (26.2)	34 (13.9)	146 (59.8)	0.955	30 (12.3)	20 (8.2)	194 (79.5)	0.636
3	82 (22.0)	82 (22.0)	208 (55.9)		40 (10.8)	38 (10.2)	294 (79.0)	
4+	74 (25.7)	48 (16.7)	166 (57.6)		28 (9.7)	24 (8.3)	236 (81.9)	
**Years of residence**								
4–	88 (26.8)	54 (16.5)	186 (56.7)	0.373	34 (10.4)	32 (9.8)	262 (79.9)	0.927
5+	132 (22.9)	110 (19.1)	334 (58.0)		64 (11.1)	50 (8.7)	462 (80.2)	

For a clearer picture of the comparison, we have merged “never,” “occasionally,” “sometimes,” “often,” “always,” “always” level 5 items into level 3, “never,” “occasionally” into “less,” “often,” and “always” into “many”.

### The overall model

**Figure 1:**
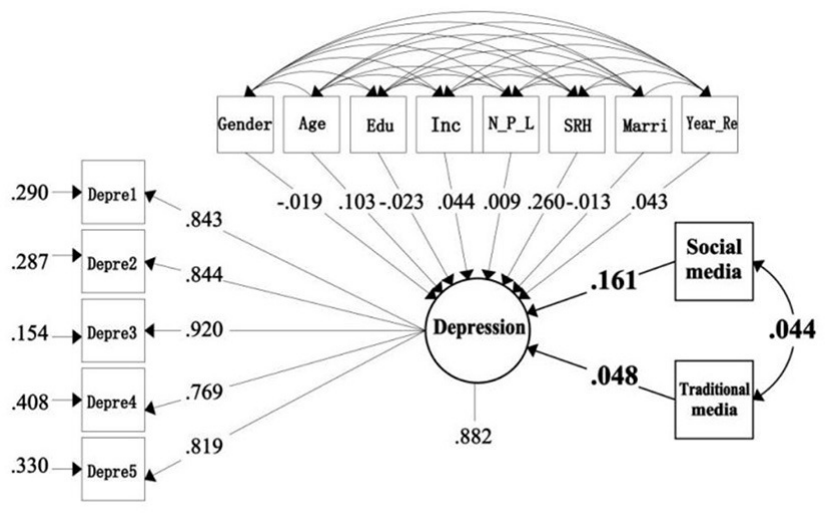
The standardized path of depression influenced by social media.

**Figure 2:**
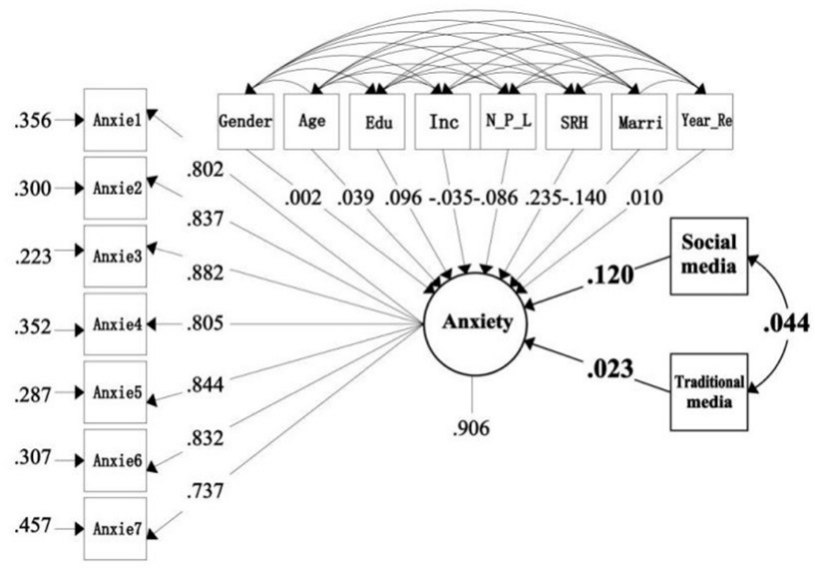
The standardized path of anxiety influenced by social media. Figure 1 and this figure were drawn by the authors.

Table 3 shows the model fitting results based on the overall sample. When gender, age, education, income, marital status, number of people living together, years of residence, and SRH were controlled, social media had a significant positive correlation between depression and anxiety at 1%level. The effects of standardization were 0.161 (*P* = 0.000) and 0.120 (*P* = 0.001), respectively However, the use of traditional media is not significantly related to anxiety and depression. In addition, we can also see that age and SRH have a significant positive effect on depression at 1% level, with standardized effect coefficients of 0.103 (*P* = 0.008), 0.260 (*P* = 0.000), and no significant correlation between other covariates and depression (Figure 1). Education and SRH had a significant positive effect on anxiety at 1% level, with standardized influence coefficients of 0.096 (*P* = 0.008) and 0.235 (*P* = 0.000), respectively. Marriage had a significant negative effect on anxiety at 1% level, with a standardized effect factor of −0.140 (*P* −0.000), and no significant correlation between the other covariates and anxiety (Figure 2).

**Table 3:** Standardization coefficient and significance of the overall model.

**Variable**		**Standardization estimate**
Depression	Independent variable	Traditional media	0.048
		Social media	0.161^***^
	Covariates	Gender	−0.019
		Age	0.103^***^
		Edu (education)	−0.023
		Inc (income)	0.044
		Marri (marital status)	−0.013
		SRH (self-rated health)	0.260^***^
		N_P_L (number of people living together)	0.009
		YEAR_RE (years of residence)	0.043
Anxiety	Independent variable	Traditional media	0.023
		Social media	0.120^***^
	Covariates	Gender	0.000
		Age	0.039
		Edu (education)	0.096^***^
		Inc (income)	−0.035
		Marri (marital status)	−0.140^***^
		SRH (self-rated health)	0.235^***^
		N_P_L (number of people living together)	−0.086^**^
		YEAR_RE (years of residence)	0.000

This table shows the results of the model fitting of social media's effects on depression and anxiety based on a holistic sample, with traditional media and other covariates added to the model for better comparison. At the same time, in order to unify the consistency of the positive and negative directions of the path, we inverted the data in the table when we processed the model. In the table, all positive values are expressed as beneficial effects on a person's mental health, and all negative values are expressed as adverse effects on a person's mental health.

*** Means significance at the 0.01 level.

** Means significance at the 0.05 level.

^*^Means significance at the 0.1 level.

We used PSM to analyze the results of structural equation models for robustness analysis and sensitivity Analyses. First, we divided the samples of residents into experimental and control groups according to the difference in social media use frequency, with the top 50% of the samples in social media use frequency as the experimental group and the rest as the control group. The gender, age, education, income, marital status, number of people living together, years of residence, and SRH are then used as disruptors through binary Logit. The model estimates the probability of each sample being grouped into the experimental group and obtains its propensity score. Match matching is then performed, matching the samples with the closest propensity values but belonging to two groups one by one.

We used the nearest neighbor matching method for matching, and the results showed that the matching success rate was 100%. The propensity score matching (PSM) effect is detected by parallel hypothesis testing. The results showed (Table 4) that the absolute values of the normalized deviation after matching were <20%, and the normalized deviation value was significantly reduced, and the matching effect was better; the *t*-test after matching was not significant (*p* > 0.05), age, Marital status, income, and self-assessment health were several variables that were significant before the *t*-test (*p* < 0.05), and the *t*-test after matching had no significance (*p* > 0.05), indicating that the matching effect is good.

**Table 4:** PSM parallel hypothesis test.

**Disturbing variable**		**Treated**	**Control**	**Standard deviation (%)**	**Reduction of standard deviation (%)**	**t**	***p***
Gender	Unmatched	1.661	1.636	5.25	33.39	0.774	0.439
	Matched	1.661	1.644	3.50		0.469	0.639
Age	Unmatched	2.483	2.688	−30.53	75.60	−4.513	0.000
	Matched	2.483	2.533	−7.45		−0.999	0.318
Edu	Unmatched	3.683	3.540	13.33	64.06	1.968	0.049
	Matched	3.683	3.633	4.79		0.643	0.520
Inc	Unmatched	4.300	4.114	12.89	93.99	1.885	0.060
	Matched	4.300	4.311	−0.77		−0.104	0.917
SRH	Unmatched	2.156	2.004	21.76	89.04	3.194	0.001
	Matched	2.156	2.172	−2.39		−0.320	0.749
N_P_L	Unmatched	3.028	3.077	−5.61	0.29	−0.824	0.410
	Matched	3.028	2.978	5.59		0.751	0.453
YEAR_RE	Unmatched	3.139	2.985	12.84	32.82	1.885	0.060
	Matched	3.139	3.239	−8.63		−1.158	0.247

The matched data were analyzed by the Average treatment effect (ATT) (Table 5), and it can be seen that the ATT effect value after matching is still significant (*p* < 0.05), i.e., the PSM analysis showed significant differences between social media use and depression and anxiety, and social media use has a positive effect on both depression and anxiety.

**Table 5:** ATT analysis.

**Disturbing variable**		**Treated**	**Control**	**Difference**	**Std. error**	**t**	***p***
Depression	Unmatched	3.162	2.696	0.467	0.092	5.074	0.000
	Matched	3.162	2.534	0.628	0.102	6.182	0.000
Anxiety	Unmatched	0.600	0.703	0.103	0.044	2.328	0.020
	Matched	0.600	0.689	0.089	0.049	1.805	0.031

### Comparison of paths among different group models

Table 6 compare model fitting results based on different age and gender samples. When covariates were controlled, the effects of social media use on depression and anxiety in the youth and female groups were significant at 1% level. The standardized effect coefficients were: youth depression 0.202 (*P* = 0.000), youth anxiety 0.200 (*P* = 0.000), female depression 0.196 (*P* = 0.000), female anxiety 0.129 (*P* = 0.008). The effects of social media use on depression in middle-age and above groups were significant at 5% level (standardized effect coefficient was 0.134, *P* = 0.015), but the effects on anxiety were not significant. The effects of male social media use on depression and anxiety were significant at 10% level, with standardized effect coefficients of 0.112 (*P* = 0.083) and 0.118 (*P* = 0.076), respectively.

**Table 6:** Comparison of path factors for different group models.

**Variable**			**Group**			
			**Age**		**Gender**	
			**39–**	**40+**	**Female**	**Male**
Depression	Independent variable	Traditional media	0.093	−0.017	0.054	0.012
		Social media	0.202^***^	0.134^**^	0.196^***^	0.112^*^
	Covariates	Gender	−0.079	0.025	–	–
		Age	−0.027	0.141^***^	0.148^***^	−0.017
		Edu	0.087	−0.075	0.019	−0.114^*^
		Inc	0.013	0.067	0.068	−0.027
		Marri	−0.089	0.081^*^	0.036	−0.128^*^
		SRH	0.147^***^	0.371^***^	0.263^***^	0.243^***^
		N_P_L	0.030	0.020	−0.013	0.050
		YEAR_RE	0.003	0.081^*^	0.051	0.020
Anxiety	Independent variable	Traditional media	−0.018	−0.024	−0.028	−0.018
		Social media	0.200^***^	0.060	0.129^***^	0.118^*^
	Covariates	Gender	0.000	0.001	–	–
		Age	0.064	0.015	0.030	0.043
		Edu	0.039	0.117^**^	0.103^**^	0.072
		Inc	−0.090^*^	0.024	−0.017	−0.048
		Marri	−0.150^***^	−0.141^***^	−0.105^**^	−0.217^***^
		SRH	0308^***^	0.184^***^	0.211^***^	0.270^***^
		N_PLT	−0.059	−0.138 ^***^	−0.048	−0.163^***^
		YEAR-RE	−0.095^*^	0.075^*^	0.005	−0.013

This table shows the effects of social media on depression and anxiety based on different age and gender samples, as in Table 3, with traditional media and other Covariates added to the model, while the data in the anxiety scale were reversed. In the table, all positive values are expressed as beneficial effects on a person's mental health, and all negative values are expressed as adverse effects on a person's mental health.

*** Represents significant at the 1% level.

** Represents significant at the 5% level.

* Represents significant at the 10% level.

The only covariate that had a significant impact on depression in the youth group were SRH, the standardized influence coefficient was 0.147 (*P* = 0.004), and the other covariates had no significant correlation with depression in the youth group. Age and SRH had a significant positive effect on depression in middle-age and above groups, and the standardized effect coefficients were 0.134 (*P* = 0.012), 0.371 (*P* = 0.000). Only age and SRH were significantly affected by depression in female groups, and the standardized influence coefficients were 0.148 (*P* = 0.001) and 0.263 (*P* = 0.000), respectively. Education, marriage, and SRH had significant positive effects on depression in male, with standardized influence coefficients of −0.114 (*P* = 0.052), −0.128 (*P* = 0.060), and 0.243 (*P* = 0.000), respectively.

The factors that had a significant effect on anxiety in the youth group were SRH, marriage, and income with standardization coefficients of 0.308 (*P* = 0.000), −0.140 (*P* = 0.000), 0.090 (*P* = 0.095), respectively. Factors that have a significant impact on anxiety in middle-age and above group were SRH, education, marriage, and the number of people living together, with standardized influence coefficients of 0.184 (*P* = 0.000), 0.117 (*P* = 0.020), −0.141 (*P* = 0.008), −0.138 (*P* = 0.010), respectively. Other covariates had no significant correlation with anxiety in middle-age and above group. The only covariates that had a significant impact on anxiety in the female group were education and SRH, and the standardized effect coefficients were 0.103 (*P* = 0.026), 0.211 (*P* = 0.000), respectively. Marriage, the number of people living together, and SRH had significant effects on male's anxiety, with standardized influence coefficients of −0.217 (*P* = 0.001), −0.163 (*P* = 0.004), 0.270 (*P* = 0.000). Other covariates have no significant correlation with male anxiety.

## Discussion

Our study explores social media use and mental health problems among Chinese urban residents during the COVID-19 outbreak in January 2021. The purpose of our research is to reveal whether the mental health of residents during the second outbreak of the COVID-19 occurred after the pandemic was largely under control in China, and whether the state of social media's impact on the mental health of residents has changed in the wake of systematic social media governance in China.

Our cross-sectional study shows that more than one-third (38.9%)of the population suffered from depression and more than one-eighth (12.61%) suffered from anxiety during the second outbreak in China in January 2021. According to the latest national sample study, the prevalence of depression in China is 6.9% (37). These findings are consistent with previous studies that public health emergencies can lead to public mental health problems (23, 38–40). But it is gratifying to note that our findings are in striking contrast to those of some scholars on the mental health of residents during the first outbreak of the COVID-19 in China in 2019 (11). These indicate that mental health problems caused by the COVID-19 outbreak are gradually being cured in China. Our study also found that different social groups have different levels of mental health, with more mental health problems among groups of young people, female, low-income, unmarried, students, poor of SRH and more of shared housing, which require special attention.

Social media is one of the main sources of information on COVID-19 (7, 8). Our study analyzed that during the second round of the COVID-19 outbreak in China, the average Chinese relied more heavily on social media. 57.7% of participants used traditional media, but up to 80.1% of participants used social media instead. The frequency of use of social media is significantly higher than that of traditional media.

Our findings are quite the opposite of previous conclusions about the effects of social media on mental health (2020) (3, 12, 13). During China's second COVID-19 outbreak in January 2021, increased use of social media significantly improved depression and anxiety among residents, outpacing the often-widely accepted factors of influence such as education, income, and age.

For the first time, the study found that the relationship between social media use and residents' mental health has changed radically since the first COVID-19 outbreak in 2019, and one of the reasons behind may stem from China's effective management of the “information epidemic” of social media. During the first outbreak of COVID-19 in China in 2019, social media became an “information epidemic,” and false information and reports about COVID-19 bombarded social media, sparking unfounded fears among many netizens (15). In addition, many citizens expressed their negative emotions on social media, such as fear and tension, which spread rapidly on social networks (41, 42). These behaviors can harm people's mental health. With the “information epidemic” seriously affecting people's mental health, China has quickly and decisively implemented a series of effective governance of the social media environment: (1) make good use of social media. On the one hand, major social media (such as Sina, Tencent, NetEase, etc.) set up “anti-epidemic” module, acting as “watch sentinel” to monitor and warn of the development of the COVID-19 epidemic, improving the speed of emergency response, so that authoritative information can be first posted on social media. At the same time, a special column has been set up to allow rumors to be detected in the first place, and social media has been used to greatly improve emergency management and service levels in the government and the public sectors. On the other hand, China vigorously promotes the positive energy of society in the social media. A series of promotion about patriotism, dedication, love, and other positive energy of the social environment has been carried out through specific films, television dramas, short videos, animations, interaction, and other forms, which vigorously consolidate the Chinese cohesion. (2) Effectively regulate social media. Both strict law enforcement and moderate tolerance of some complaints are consolidated, so that social media can act as the smooth channels to understand people's feelings, to resolve grievances of the people, and as an important helper of the government to find and solve problem. For those who maliciously produce and disseminate rumors, legal responsibility will be investigated.

Our study also found some interesting conclusions. First, our study found that the impact of traditional media in China is clearly decreasing: the frequency of traditional media use is much lower than that of social media use, and it is not significantly relevant to residents' mental health. What's more, we found that the relationship between social media use and mental health varied significantly among different groups, with women and young people having a stronger correlation between mental health and social media use, while men, middle-aged and above having a weaker correlation. We recognized that women and young people are such the groups as relatively suffer from serious mental health problems, and it is precisely such the groups whose mental health is more affected by social media. To deal with this situation, special attention and help from all levels of governments and relevant departments in China are needed for young people and female groups. The social media environment should continue to be optimized, especially to focus on more information on the care and encouragement of female and youth groups.

There are some limitations to this study. First, although we used the “quasi-experimental” PSM to conduct causal verification and robustness analysis, and tried to analyze the causal relationship between social media and residents' mental health as much as possible, our study is based on cross-sectional data, and it's difficult to accurately clarify the casual relationship, and it requires a follow-up longitudinal study.

In addition, the survey was conducted online, so there may be some bias in the representation of the sample, such as the low participation of older persons, which may affect the results of the assessment. But the advantage of web-based investigations is that they can get data quickly in the event of another emergency. Finally, although we control as many covariates as possible, we cannot rule out residual mixing due to unmeasured factors.

## Conclusion

Our research showed that the mental health problems caused by the second outbreak of the COVID-19 in China in January 2021 are less serious than the first outbreak in 2019. Simultaneously, China's social media environment has been optimized. During the second outbreak, the relationship between social media use and residents' health has changed radically, with more social media use significantly relevant to fewer mental health problems, especially among women and young people. Our research found that during the large public health outbreaks, there exists heterogeneity not only in public mental health problems, but also in the degrees of correlation between social media and residents' mental health. Our conclusions provide a basis and clue for the subsequent precise intervention of people's mental health problems during public health events. We, therefore, call for the need to continuously optimize the social media environment, with a particular focus on more information on caring, mentoring and encouraging women, youth, low-income groups, and other relatively vulnerable groups.

## Data availability statement

The original contributions presented in the study are included in the article/supplementary material, further inquiries can be directed to the corresponding author.

## Author contributions

ZZ and HC: conceptualization and resources. ZZ: writing—original draft preparation, software, and funding acquisition. ZZ, HC, and NS: methodology, validation, data curation, and visualization. HC and YC: writing—review and editing. HC: project administration and supervision. All authors have read and agreed to the published version of the manuscript. All authors contributed to the article and approved the submitted version.
